# Extracellular vesicles arising from apoptosis: forms, functions, and applications

**DOI:** 10.1002/path.6138

**Published:** 2023-06-09

**Authors:** Christopher D Gregory, Michael P Rimmer

**Affiliations:** ^1^ Centre for Inflammation Research Institute for Regeneration and Repair, University of Edinburgh Edinburgh UK; ^2^ Centre for Reproductive Health Institute for Regeneration and Repair, University of Edinburgh Edinburgh UK

## Abstract

Extracellular vesicles (EVs) are lipid bilayer‐enclosed subcellular bodies produced by most, if not all cells. Research over the last two decades has recognised the importance of EVs in intercellular communication and horizontal transfer of biological material. EVs range in diameter from tens of nanometres up to several micrometres and are able to transfer a spectrum of biologically active cargoes – from whole organelles, through macromolecules including nucleic acids and proteins, to metabolites and small molecules – from their cells of origin to recipient cells, which may consequently become physiologically or pathologically altered. Based on their modes of biogenesis, the most renowned EV classes are (1) microvesicles, (2) exosomes (both produced by healthy cells), and (3) EVs from cells undergoing regulated death by apoptosis (ApoEVs). Microvesicles bud directly from the plasma membrane, while exosomes are derived from endosomal compartments. Current knowledge of the formation and functional properties of ApoEVs lags behind that of microvesicles and exosomes, but burgeoning evidence indicates that ApoEVs carry manifold cargoes, including mitochondria, ribosomes, DNA, RNAs, and proteins, and perform diverse functions in health and disease. Here we review this evidence, which demonstrates substantial diversity in the luminal and surface membrane cargoes of ApoEVs, permitted by their very broad size range (from around 50 nm to >5 μm; the larger often termed apoptotic bodies), strongly suggests their origins through both microvesicle‐ and exosome‐like biogenesis pathways, and indicates routes through which they interact with recipient cells. We discuss the capacity of ApoEVs to recycle cargoes and modulate inflammatory, immunological, and cell fate programmes in normal physiology and in pathological scenarios such as cancer and atherosclerosis. Finally, we provide a perspective on clinical applications of ApoEVs in diagnostics and therapeutics. © 2023 The Authors. *The Journal of Pathology* published by John Wiley & Sons Ltd on behalf of The Pathological Society of Great Britain and Ireland.

## Introduction

Apoptosis, the most renowned and, in cellular, molecular, and pathophysiological terms, the best‐understood cell death programme, has been described aptly as ‘controlled demolition at the cellular level’ [1]. This reflects the regulated ‘self‐disassembly’ of individual cells that culminates in their phagocytic removal, usually with minimal collateral tissue damage. Originally named by the pathologists Kerr, Wyllie, and Currie using classically morphological criteria such as cell shrinkage, chromatin condensation, and cellular fragmentation along with an appreciation of its regulated nature [2], apoptosis has been defined more recently in biochemical terms as ‘a caspase [cysteine protease]‐dependent subroutine of regulated cell death’ [3]. Cells are estimated to engage their apoptosis programme in humans at the rate of around 1 million per second and often (though not always) fragment into phospholipid bilayer‐bound structures known commonly as apoptotic bodies (ApoBDs). These represent a subset – typically of relatively large size range – of the spectrum of extracellular vesicles (EVs) produced by apoptotic cells (ApoEVs). The term extracellular vesicle is now accepted as a generic description of subcellular, membrane‐delimited particles, ranging from tens of nanometres to several micrometres in size. EVs can play key roles in intercellular communication through the carriage and transfer of diverse bioactive cargoes such as nucleic acids, proteins, lipids, and metabolites that reflect their cells of origin [4]. As recommended by the International Society for Extracellular Vesicles, the generic term ‘EV’ encompasses exosomes, microvesicles, ectosomes, oncosomes, and other putative classes of EVs that have been described – in addition to ApoEVs – using various criteria, including modes of biogenesis, size ranges, and cargoes [5].

Apoptosis is widely accepted as a process in which EV production is increased. Indeed, ApoEVs of various sizes and organellar constituents have long been held as a defining feature of apoptosis [2, 6]. However, just as the active communicative nature of apoptosis has often been overlooked, knowledge of the biology of ApoEVs lags behind that of EVs produced in contexts unrelated to cell death. EV research has developed substantially, especially over the last two decades, and the reader is referred to several excellent reviews that summarise historical progress, nomenclature, and current knowledge of EV biogenesis, the structure and function of EVs, and clinical aspects of general EV biology [4, 5, 7, 8, 9, 10, 11, 12, 13, 14]. The purpose of this review is not only to outline what is currently known about the production, characteristics, and properties specifically of ApoEVs but also to highlight critically the significant gaps in our knowledge of them. A perspective on the pathophysiological and translational aspects of ApoEVs is also provided.

## Concerning EVs, ApoEVs, and ApoBDs

### General aspects of EVs

**Figure 1:**
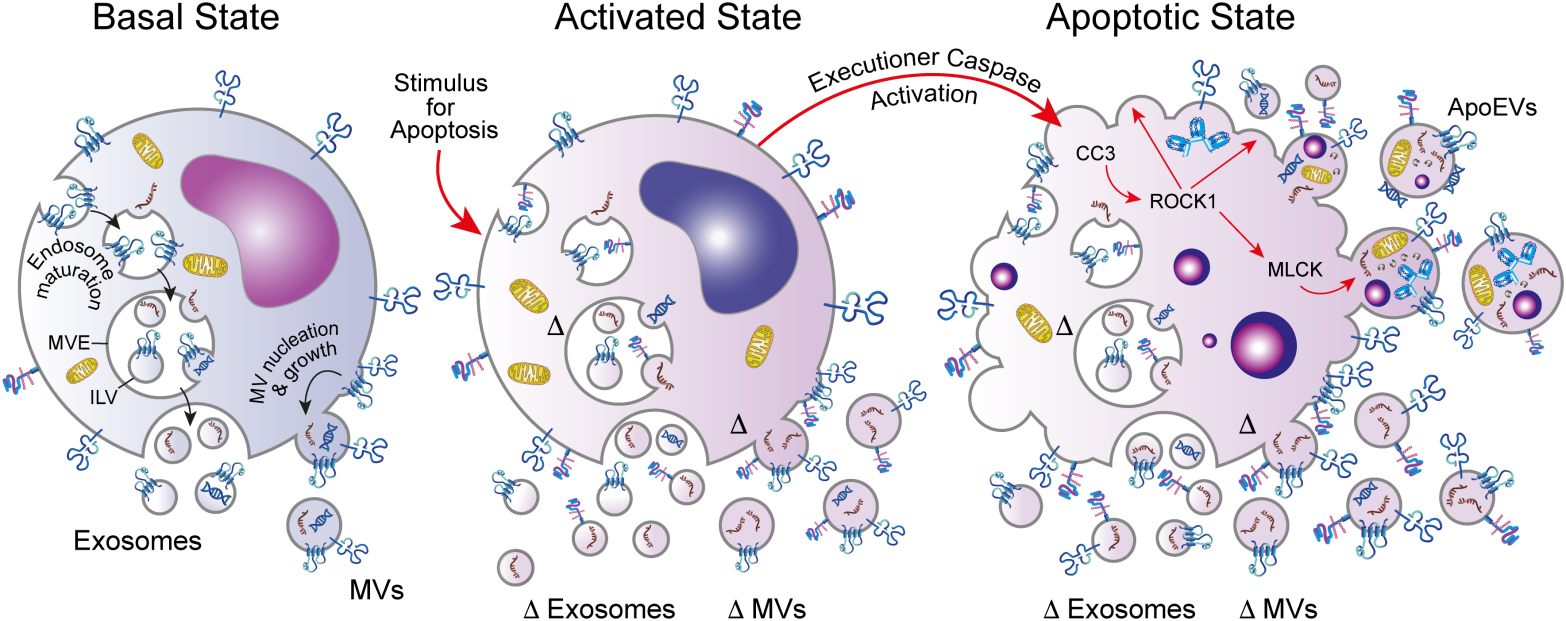
Schematic representation of EV formation and ApoEV heterogeneity. EV populations produced under basal conditions (left) comprise, at least, mixtures of exosomes and MVs with overlapping sizes and cargoes. In stressed or activated cells that have received a stimulus for apoptosis (centre), qualitative and quantitative changes (Δ) in exosomes and MVs are anticipated. In the apoptotic state (right), activation of executioner caspases, notably cleaved caspase 3 (CC3), promotes bleb formation through activation of ROCK1, leading to MLCK activation and production of ApoEVs with diverse cargoes, including organelles, proteins, and nucleic acids. Accumulation of exosomes and MVs, along with ApoEVs, generates complex heterogeneity in ApoEV populations. See text for details of EV biogenesis pathways. Little is currently known about the biogenesis of putative ApoEV subtypes, which are depicted here as MV‐like but may also be generated through alternative pathways. ApoEV biogenesis can be regulated by PANX1 and PlexB2 and may also involve protrusions such as apoptopodia. ILV, intraluminal vesicle; MVE, multivesicular endosome.

Based on fundamental differences in their main modes of biogenesis, EVs can be broadly divided into two major categories: (1) exosomes and (2) ectosomes, the latter also known as microvesicles (MVs, Figure 1; it is also noteworthy that an increasing variety of extracellular particles, some of which are not classified as EVs, are known to be released by a variety of cell types [13]). Exosomes are EVs of small, spherical size (commonly 30–150 nm in diameter), produced within the endosomal system. It is worth noting, however, that the EV literature abounds with publications that use this designation as a generic descriptor of EVs. To add to the confusion, the term exosome is also used to describe conserved intracellular protein complexes that process RNA in eukaryotic cells [15].

Inward budding (away from the cytosol) of endosome membranes results in the production of intraluminal vesicles and transforms endosomes into multivesicular bodies, otherwise known as multivesicular endosomes (MVEs). Subsequent fusion with the plasma membrane permits release of the intraluminal vesicles into the extracellular space as exosomes (Figure 1). Highly significant molecular players in the biogenesis of exosomes are the endosomal signalling complex related to transport (ESCRT) protein complexes that mediate the fission of endosomal buds to produce intraluminal vesicles and that are often required to drive the inward endosomal budding process that precedes fission [8]. Additional proteins, such as ALG‐interacting protein X, TSG101 and the tetraspanins (four transmembrane domain‐containing proteins) CD9, CD63, and CD81 are also frequently highlighted among exosome cargoes, but it should be noted that these proteins, among isolated EV preparations, do not appear to be exosome‐specific [16]. The second major class of EVs, the MVs, also form through budding away from the cytosol, but, in contrast to exosomes, they are produced at the plasma membrane, probably more rapidly than exosomes (Figure 1). Typically, MVs are 50–1,000 nm in diameter but can also reach several micrometres in size, as in the case of large oncosomes (MVs of 1–10 μm produced by tumour cells) and ApoBDs [8, 17, 18]. The reader is referred to excellent reviews on the evolving molecular mechanisms of exosome and MV biogenesis (e.g. [8, 11, 14, 17]).

### ApoEVs and ApoBDs

ApoEVs are thought mainly to represent a subclass of MVs since, just as the MVs of healthy cells, most ApoEVs seem likely to be produced by plasma membrane outward budding. (Throughout this review, we use the term healthy cells loosely to refer to cells that are not dying or being signalled to die by apoptosis or any other form of cell death.) ApoEV production is defined as occurring only in cells that have activated their apoptosis programme. Since apoptosis can be triggered by multiple and diverse stimuli, ApoEVs that are produced as a specific consequence of apoptosis must be carefully discriminated from EVs generated by those signalling pathways that are proximal to the activation of the apoptosis execution machinery *per se*, including cell stress signals, along with EVs that may be produced constitutively by the cells in question. Thus, just as EVs produced from healthy cells are heterogeneous, populations of EVs produced by cells triggered into apoptosis are almost certain to contain ApoEVs contaminated with exosomes, MVs, and potentially other types of EVs whose production is not specifically dependent upon the apoptosis programme (Figure 1). For these reasons, the extent of ApoEV diversity remains poorly defined; most of our current knowledge of ApoEVs pertains to ApoBDs, which, in addition to their historic morphological features, have largely been discriminated by size, density, and flow cytometric properties following their release from cells showing distinct, classical characteristics of apoptosis, including fragmentation [19, 20, 21, 22].

Sometimes considered to include whole, morphologically defined apoptotic cells, ApoBDs are more generally regarded as MVs produced during the regulated dismantling of many different types of cells undergoing apoptosis (many studies fail to discriminate between originator apoptotic cells and the relatively large ApoBDs they produce; some use the terms apoptotic cell and apoptotic body interchangeably). Essentially a subset of ApoEVs of relatively large size (often 1–5 μm in diameter, but may be larger or smaller [23]), ApoBDs often contain organelles of healthy morphological appearance, including mitochondria, ribosomes, and endoplasmic reticulum, as well as distinct nuclear fragments [2]. These relatively large ApoEVs are not a feature of all types of apoptotic cells; in certain cells they have been shown to be produced through a stepwise process of apoptotic cell disassembly in association with fine plasma membrane protuberances of the dying cells described as microtubule spikes, apoptopodia, and beaded apoptopodia [21, 24, 25]. The significance of these specific processes remains largely unknown, but the production of ApoBDs – and indeed all ApoEVs, large and small – has functional implications for translating the meaning of apoptosis.

Apoptotic cell blebbing at the plasma membrane and fragmentation into ApoBDs is dependent on the Rho‐associated coiled‐coil‐containing protein kinase 1 (ROCK1), which becomes constitutively activated following removal of its C‐terminal inhibitory domain by cleaved caspase 3 [26, 27]. Inhibition of ROCK1 activation has been shown to suppress the formation and phagocytosis of ApoBDs in parallel by certain types of phagocytes *in vitro*, although apparently not primary macrophages [28, 29]. Therefore, the breakdown of apoptotic cells into ApoBDs may facilitate apoptotic‐cell clearance by phagocytes (efferocytosis). This appears also to be the case for apoptotic monocytes, which require caspase 3 cleavage of the cell surface receptor plexin B2 for both effective dismantling into ApoBDs (via apoptopodia) and for efficient efferocytosis by alveolar macrophages and by alveolar epithelial cells [30]. The importance for clearance of the ‘bite‐sizing’ fragmentation aspect remains unclear, however, because caspase‐induced activation of ROCK1 is required for multiple characteristic morphological features of apoptosis, including cell contraction and membrane blebbing in addition to ApoBD formation. It is noteworthy that mice in which ROCK1 was made inactive by mutation of its kinase‐activating caspase cleavage site were found to be ostensibly normal in terms of their non‐phlogistic basal apoptosis activities. By contrast, tissue‐wide diethylnitrosamine‐induced apoptosis in the livers of wild‐type mice became switched in the caspase‐non‐cleavable ROCK1 mutant animals to a necrosis‐like cell death and associated sterile inflammation. Intriguingly, this was associated with suppression of the development of diethylnitrosamine‐driven hepatocellular carcinoma [31]. It seems that ApoEV production in the dismantling of apoptotic cells that ensures efficient, non‐phlogistic clearance depends markedly on cell and tissue context as well as multiple molecular mechanisms. As we will discuss later, the most acclaimed, and arguably most important, component of the apoptotic cell's plasma membrane that enables intercellular communication is the externalised anionic phospholipid, phosphatidylserine (PtdSer). Exposed PtdSer may be similarly important in the functions of ApoEVs.

## The apoptosis secretome and ApoEV formation

It is well established that apoptosis engenders a broad spectrum of microenvironmental and systemic responses from healthy cells [32], the most famous being the activation of macrophages for swift clearance of apoptotic cells and ApoBDs, normally in a non‐phlogistic fashion. Leakage of potentially pro‐inflammatory and immunostimulatory intracellular components is prevented through retention of plasma membrane integrity prior to efferocytosis. Biologically active components of free apoptotic cells include secreted soluble factors, plasma membrane components, and ApoEVs. Among soluble factors are mononuclear phagocyte chemoattractants such as the nucleotides adenosine triphosphate (ATP) and uridine triphosphate [33], immune modulators like transforming growth factor (TGF)‐β1 and interleukin (IL)‐10 [34, 35], the granulocyte ‘keep out’ signal, lactoferrin [36], and a range of metabolites that alter several gene expression programmes in phagocytes, including anti‐inflammatory, tissue‐repair, and pro‐survival pathways [37]. An important mechanism for the release of some of the relatively smaller soluble factors (<~1 kDa in size) from apoptotic cells, including the aforementioned nucleotides and metabolites, involves Pannexin 1 (PANX1) channels in the plasma membrane, which are opened through the action of the executioner caspases 3 and 7 [37, 38].

Like the apoptosis programme itself, ApoEV production is a consequence of regulated, energy‐dependent processes. ApoEVs and their cargoes constitute an important facet of the secretome and downstream biological signalling properties of apoptotic cells. ApoEVs may serve not only to expand the ‘reach’ of the apoptotic cell surface but also to carry (and protect from degradation) manifold cargoes ranging from whole organelles to small metabolites possessing diverse potential bioactivities. For example, as we will detail in the next section, ApoEVs carry functional chemoattractants and diverse immunomodulatory cargoes. It is inherently obvious that cargo capacity is limited by EV size with small EVs being physically restricted from carrying organelles [39]. Intriguingly, ApoBD size appears to be governed by PANX1, although the mechanism is not yet defined [40].

### MV‐related production mechanisms

The genesis of ApoEVs is known to encompass two features in common with MV production from healthy cells (but with different underlying mechanisms): (1) plasma membrane phospholipid changes and (2) cytoskeletal alterations leading to microvesicular budding directly from the plasma membrane of the dying cell. Exosomal pathways to EV production may also be active during (or leading up to) apoptosis [41]. Aspects of the biogenesis of MVs and of ApoBDs have been reviewed recently [8, 22], but detailed molecular mechanisms remain unclear (see also Figure 1). In brief, ApoBDs originate from the loss of phospholipid asymmetry in the plasma membrane, which appears to promote membrane bending, facilitating vesicular budding from the cell surface. The latter is often described in relation to the dynamic blebbing of the cell surface, which is a classic characteristic of apoptosis when observed in real time by light microscopy. Loss of plasma membrane asymmetry – most remarkably externalisation of the anionic phospholipid, PtdSer – occurs in apoptosis as a result of irreversible inhibition, through caspase activity, of the ATP‐dependent aminophospholipid translocase (‘flippase’) ATP11 (which otherwise actively flips PtdSer to the inner plasma‐membrane leaflet in healthy cells), along with caspase‐mediated irreversible activation of the lipid scramblase, Xkr8. In other contexts, caspase‐independent, reversible PtdSer exposure in plasma membranes can occur, either through the activity of the TMEM16F scramblase or via phosphorylation of Xkr8 [42]. Of particular note, the ubiquitously expressed TMEM16F is required for MV production by platelets [43]. Taken together, this evidence supports the view that multiple signalling pathways, in both the proximal activation phase and the execution phase of apoptosis, rapidly redistribute phospholipids from the inner to the outer plasma membrane leaflet to initiate plasma membrane blebbing and ApoEV formation.

As indicated earlier, dynamic blebbing of the plasma membrane is dependent upon effector caspase cleavage and activation of ROCK1, which in turn activates myosin light chain kinases (MLCK) and drives actomyosin contractility. It is likely that bleb genesis and growth initially progresses as in cytokinesis and cell migration, wherein the plasma membrane detaches from the underlying actin cortical cytoskeleton as increased hydrostatic pressure by the actomyosin system propels bleb expansion [44]. Cytosol flows into the growing bleb, as do lipids into its membrane, and the cortical cytoskeletal structure subsequently reforms beneath the membrane. In apoptosis, although bleb retraction is common (just as in cytokinesis and cell migration), many blebs are released as ApoEVs/ApoBDs [45]. MV formation in healthy cells is also triggered by MLCK activation and myosin light chain phosphorylation. The mechanism is caspase‐independent: the guanosine triphosphate‐binding protein, ADP‐ribosylation factor 6 (ARF6), activates phospholipase D, which results in the recruitment of extracellular signal‐regulated kinase (ERK) to the plasma membrane. ERK then phosphorylates MLCK which drives MV growth [46]. It seems plausible that this chain of events may also be capable of stimulating ApoEV production since, during apoptosis, calpain and gelsolin are known to be activated, the former via release of Ca^2+^ ions from the endoplasmic reticulum, the latter by caspase 3 cleavage and required for the generation of typical morphological features of apoptosis [47]. These processes can also activate MLCK phosphorylation by ERK with subsequent MV production [48, 49].

As for all MVs, the mechanisms underlying the final stages of ApoEV release are as yet poorly understood. It seems significant that components of the contractile protein machinery such as phosphorylated MLCK2 are associated with the necks of budding MVs, suggesting a ‘drawstring‐like’ fission mechanism [50]. Additional intrinsic fission mechanisms (involving ESCRT proteins) akin to those involved in vesicle release into the MVE lumen may also contribute to the liberation of ApoEVs. Extrinsic mechanisms, including shear stress, tissue mechanics, and ‘pinching off’ by phagocytes, are also likely to be important processes in ApoEV release [8, 22]. In certain cells, as we have discussed, the final stages of ApoEV release occur via the formation of distinct protuberances such as apoptopodia.

## Cargoes of ApoEVs

The cargoes of free ApoEVs were initially observed morphologically: Early transmission electron microscopic observations noted the overtly heterogeneous nature of ApoBDs with respect to nuclear fragments and cytoplasmic organelles such as lysosomes, endoplasmic reticulum, and mitochondria displaying well‐preserved, regular features like those of healthy cells [2, 6]. In this respect, ApoBDs may appear to resemble miniatures of the originator apoptotic cells. Flow cytometric analyses have extended this picture and confirmed that ‘subsets’ of ApoBDs can be distinguished on the basis of the presence or absence of nuclear and/or mitochondrial material [20, 51]. Proteomic, genomic, and other studies have shown that ApoEVs carry diverse cargoes representative of their apoptotic cells of origin, including surface and cytosolic proteins, RNA species, and DNA [8, 40, 52, 53]. However, the extent to which cargo loading into ApoEVs occurs through active targeting mechanisms is not yet understood. In their original definition of apoptosis, Kerr and colleagues noted the variation in sizes and ultrastructural contents of ApoBDs and proposed that ‘the content of an apoptotic body depends on the cellular constituents that happened to be present in the cytoplasmic protuberance that gave rise to it’ [2]. In addition to this stochastic loading, evidence has emerged suggesting that the cargo composition of ApoEVs may also be determined by regulated targeting mechanisms. For example, a flow cytometric study found >90% of ApoBDs that contained RNA contained no DNA, while RNA was absent from most DNA‐containing ApoBDs [54]. Furthermore, vesicular trafficking appears to regulate the formation of the apoptopodia and beaded apoptopodia that can be important for ApoEV production from some cell types [25]. In the case of healthy cells, delivery of cargoes to EVs is dependent on both the cell type and the biogenesis pathways of different classes of EVs. Markers of both exosome and MV biogenesis as well as (partially) discriminatory cargoes have been reported for both of these EV classes [8, 55, 56]. Intriguingly, recent work shows that PANX1 channels negatively regulate the loading of ApoBDs with cargoes derived from the nuclei of apoptotic cells [40]. Further investigations are required to advance our knowledge of the molecular cell biology of ApoEV biogenesis. We suggest that a combination of regulated translocation with stochastic mechanisms underlies the cargo composition of ApoEV populations.

### DNA and associated cargoes

Although the cargoes of ApoEV populations have been analysed in detail, especially in terms of their organellar, plasma membrane, proteomic, and genetic composition, generic diagnostic features of free ApoEVs have not been forthcoming, with the exception of the well‐known fragmented nuclear and characteristically cleaved genomic DNA (gDNA) contents of some ApoBDs [51, 57]. Microtubules enable nuclear shrinkage during apoptosis [58], and caspase‐mediated cleavage of nuclear lamins allows ROCK1‐dependent actomyosin activation to drive nuclear disintegration and to translocate nuclear and DNA fragments into apoptotic blebs, some of which become ApoBDs [26, 59]. As well as gDNA, blebs of apoptotic cells and ApoEVs contain internucleosomal histones [52, 58, 60]. Ribosomes, fragments of endoplasmic reticulum, nuclear RNAs, and ribonucleoprotein aggregates also translocate into surface blebs and ApoEVs [45, 61, 62, 63, 64, 65]. These cargoes all constitute common autoantigens, and dysfunctional processing of ApoEVs is closely linked to autoimmune disease pathogenesis (see following discussion). Related to these observations, splicing factors (splicing proteins and small non‐coding RNAs) have been demonstrated selectively in ApoEVs from glioblastoma cells [66].

Although common, DNA cargo is not a distinguishing property of ApoEVs since, among host‐derived DNA species, single‐stranded DNAs, double‐stranded gDNA, and mitochondrial DNA are all released in association with EVs independently of apoptosis by diverse cell types, strikingly by tumour cells [67]. Importantly, double‐stranded DNA representing the whole genome can be carried by EVs and can be used to identify mutations characteristic of the originator tumour cells [68, 69]. EV DNA may be protected from DNAse activity within the EV lumen or may be exposed on its surface [70]. Recent work has reported that double‐stranded DNA is recruited in healthy tumour cells to MVs by ARF6 in association with the cytosolic DNA sensor, *c*yclic guanosine monophosphate‐*A*MP *s*ensor (cGAS). Increased amounts of DNA per vesicle were found in MVs from aggressive, metastatic, as compared to non‐metastatic, melanoma lines [71]. Exosome and exosome‐like pathways of DNA loading have also been proposed [55, 67], but it is unknown whether these are operational in apoptotic cells. Circulating DNA in plasma of prostate cancer patients has been reported to be concentrated in large EVs (1,000–5,500 nm in diameter) [72], but whether ApoBDs contribute to large EVs in the circulation has not been investigated. Earlier work, however, suggested that prostate cancer cell lines released gDNA in association with all factions of EVs (based on size and density): exosomes, MVs, and ApoBDs. Cargo gDNA sequences of all three EV classes were found to include *TP53* and *PTEN*, genes that are frequently mutated in prostate cancer [73].

### Membrane constituents

Delimiting ApoEV membranes often reflect the plasma membrane topology of their apoptotic cell of origin with respect to phospholipid exposure, membrane protein, and glycan composition. Thus, ApoEVs are frequently characterised as having high levels of exposed PtdSer, although some are reported as low in this regard [23]. PtdSer exposure is also a feature of certain EVs from healthy cells. Furthermore, like apoptotic cells, EVs can also expose the aminophospholipid phosphatidylethanolamine [74]. ApoEVs are also likely to carry surface proteins indicative of the plasma membrane of their cell of origin. These may include lineage‐specific markers such as CD3 (T cell) and SiglecF (alveolar macrophage), as well as commonly expressed proteins such as class I MHC, integrins, and others [19, 20, 52, 55]. In addition, ApoEVs display surface molecular patterns that associate with PtdSer exposure and C1q binding sites and can be revealed through cross‐reactivity with anti‐LPS antibodies [75]. As yet of unknown significance, it is tempting to speculate that such motifs may be related to the capacity of ApoEVs to bind microbe‐interactive target cell receptors, such as pattern recognition receptors and scavenger receptors that are well known in efferocytosis [76]. It is known (though not well detailed) that the carbohydrate content of the cell membrane becomes altered during apoptosis, and such changes appear to be important for efferocytosis [77, 78, 79]. Of note, it has been reported that glycosaminoglycans disappear from apoptotic cell surfaces in concert with the exposure of PtdSer [80]. EV populations vary in their glycan profiles, which affects their biodistribution and uptake by target cells [81]. The inclusion of glycosylated molecules in the encapsulating membranes of small EVs undoubtedly contributes significantly to their size, charge, and capacity to interact with target cell surfaces [39, 82]. One investigation reported two types of ApoEVs based on differences in their glycocalyx: one high in immature, mannose‐rich glycoepitopes derived from the endoplasmic reticulum, the other derived from sialidase activity at the plasma membrane. Both ApoEV types reflected the effector caspase‐activated sialidase activity that is generated in apoptosis, but, intriguingly, the endoplasmic reticulum‐derived ApoEVs were preferentially cleared by macrophages [83]. Recent work suggests that certain types of ApoEVs expose a specific pattern of sugars, namely galactose and N‐acetylgalactosamine [84], which distinguish them from healthy cell‐derived EVs. Further investigations are needed to dissect the relationship between glycosylation patterns and biological activities of ApoEV subtypes.

## Cell targeting by ApoEVs

**Figure 2:**
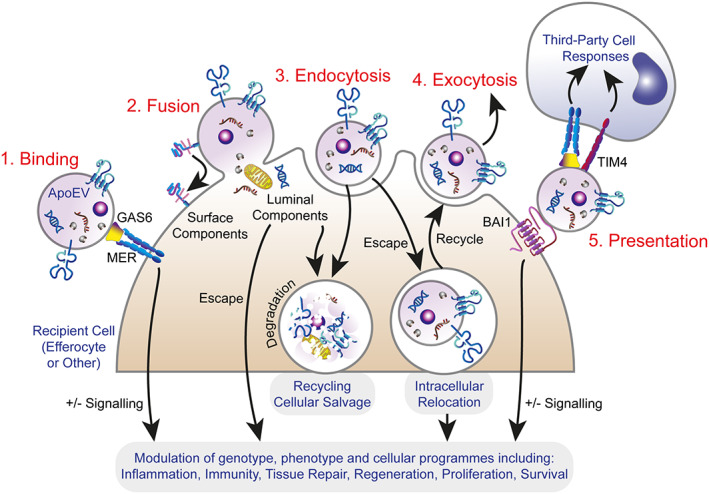
Schematic summary of theoretical mechanisms through which ApoEVs influence recipient cells. ApoEVs are likely to interact with recipient cells (either efferocytes or other cell types which do not display efferocytic activity) through multiple mechanisms: (1) Binding to surface receptors on recipient cells, here exemplified by MER interacting with ApoEVs through the PtdSer‐binding protein GAS6; (2) fusion between the delimiting membrane of the ApoEV and the plasma membrane of the recipient cell, which may cause acquisition of surface components (such as protein receptors or lipids) of the ApoEV by the recipient cell membrane and/or release of ApoEV luminal contents into the cytosol of the recipient cell; (3) uptake of ApoEVs through endosomal processes: phagocytosis, micropinocytosis, and clathrin‐, caveolin‐, and lipid raft‐mediated endocytosis. Endocytosed ApoEVs and their cargoes may be targeted for degradation and may escape for relocation in the recipient cell (for example to the nucleus); (4) intact ApoEVs may be recycled to the extracellular milieu through exocytosis; (5) recipient cell‐bound ApoEVs (here illustrated entirely speculatively by the PtdSer receptor BAI1) may present ligands to receptors (such as the PtdSer receptor TIM4 and GAS6‐engaging MER) of third‐party cells. Note that recognition and binding of ApoEVs, either to recipient or third‐party cells, may occur through diverse mechanisms, both PtdSer‐dependent and ‐independent. Lysosomal degradation of ApoEVs and their cargoes leads to recycling of apoptotic cell components through cellular salvage pathways in the recipient cells. ApoEV receptor signals and ApoEVs/cargoes that escape degradation have the potential to change fundamentally the status of recipient and third‐party cells. Molecular mechanisms remain largely unknown.

It is well established that EVs are powerful vehicles of intercellular communication, capable of profoundly altering the biology of cells with which they interact. Through transfer of biologically active cargoes, including nucleic acids and proteins and perhaps also organelles, EVs can endow recipient cells with activities of their cells of origin, either in the local tissue environment or at distant sites. Emerging evidence indicates that ApoEVs are no exception, extending the legacy and communicative properties of apoptosis in regulating tissue homeostasis. Mechanisms underlying the modes of interaction of EVs (of all subtypes) with recipient cells and of subsequent downstream cargo processing remain underdeveloped, although novel approaches that are generating valuable information are on the rise (e.g. [85]). Relevant mechanisms for healthy cell‐derived EVs have been reviewed elsewhere [13, 86, 87] and may be summarised as (a) EV engagement with the recipient cell surface, followed by (b) EV or EV cargo uptake, and subsequently (c) downstream EV and cargo processing and recipient cell responses (Figure 2). EV interaction with the recipient cell surface may activate response signalling in the recipient cell in the absence of uptake or lead either to transfer of EV luminal cargoes directly into the cytosol through membrane fusion or to take up whole EVs by endosomal processes. Most EVs appear to enter the endosomal compartment wherein their cargoes are recycled via lysosomal degradation. A minority of EV cargoes avoid degradation and enter the cytosol. Some EVs are reported to remain intact in the endosomal compartment and are rereleased [88] (Figure 2). Current knowledge of the modes of cellular targeting, intracellular processing mechanisms and of the range of functions of ApoEVs once again lags behind our understanding of the processing and functional attributes of EVs from healthy cells. Accumulated details of the molecular mechanisms underlying the responses of phagocytes to apoptotic cells, however, provide a useful platform on which research into mechanisms of ApoEV functions can be further developed.

### ApoEV targeting based on efferocytosis mechanisms

As we have seen, the cargoes of ApoEVs are at least as diverse as those of healthy cell‐derived EVs. Classical characteristics of apoptosis are (a) frequently, fragmentation of dying cells into ApoBDs and (b) engulfment of apoptotic cells and bodies – often referred to as efferocytosis or ‘corpse clearance’ – by neighbouring healthy cells of multiple lineages acting opportunistically as efferocytes or by ‘professional’ mononuclear phagocytes, most commonly tissue macrophages [89, 90]. In developmental and tissue homeostatic contexts, efficient efferocytosis provides a safe intracellular haven for the potentially pro‐inflammatory and tissue‐damaging effects of apoptotic‐cell constituents, such as proteolytic enzymes and DNA, thereby militating against autoimmune and inflammatory pathologies. Most of our knowledge of efferocytosis relates to the safe, swift clearance of apoptotic cells themselves (the corpses), but there seems little doubt that, in many of the experimental approaches, the interactions of apoptotic‐cell populations with phagocytes also include contributions from ApoBDs and other ApoEVs, even though they were not specifically characterised. In molecular terms, much is known about the sensing, phagocytic, and regulatory responses underlying corpse clearance (recently reviewed in [32, 91, 92, 93]). For example, exposed PtdSer of apoptotic cells is a key ‘eat‐me’ signal for phagocytes and helps drive their anti‐inflammatory responses. Testament to the importance of this signal is the spectrum of PtdSer receptors that have been shown to be deployable for corpse clearance. These include secreted proteins such as MFG‐E8 (lactadherin), GAS6, and Protein S, which couple exposed PtdSer to phagocyte surface receptors, as well as direct PtdSer receptors, for example TIM4, BAI1, Stabilin 2, and CD300b, which are integral to the phagocyte plasma membrane [92]. It should be noted that PtdSer exposure is insufficient for phagocytosis [94] and the loss of ‘do not eat‐me’ signals such as CD47 in the CD47‐SIRPα axis, which otherwise prevents the phagocytosis of healthy cells [95], may be important for ApoEV targeting. Other receptors involved in corpse clearance include β_3_ integrins, scavenger and pattern recognition receptors, such as CD36 and CD14, thrombospondin, and calreticulin [96]. Uptake of ApoBDs by mesenchymal stem cells (MSCs) has been reported to require the integrin α_v_β_3_ [97]. Additional molecules known to contribute to efferocytosis of apoptotic cells have also been shown to mediate uptake of ApoEVs. Thus, the hyaluronate receptor CD44, known to regulate efferocytosis of apoptotic granulocytes [98], was later reported to mediate the binding and immunosuppression of ApoEVs by dendritic cells [99]. Natural IgM antibodies, which have opsonizing activity for apoptotic cells [100], have also been shown to opsonize ApoEVs over a broad size range of ~500 nm to 5 μm. IgM‐binding ApoEVs appear to be produced relatively late in the apoptosis programme and have been proposed as a clearance mechanism that limits inflammation [101]. Other opsonins for apoptotic cells which have potential but unproven roles in ApoEV targeting and uptake include complement components such as C1q and C3b, pentraxins, and the collectins MBL, SP‐A, and SP‐D [96].

By extrapolation, it seems reasonable to expect that at least some classes of ApoEVs (e.g. the relatively large ApoBDs) will prove to utilise their surface structures, which reflect their apoptotic cell origin, to interact with known target cell receptors. While the results of extensive, formal investigations on the specific molecular mechanisms of ApoEV interaction, uptake, and cargo processing are awaited, it is noteworthy that certain of the receptors for apoptotic cells – for example TIM4, MFG‐E8, and α_v_β_3_ – are already recognised as receptors for EVs from healthy cells [102, 103]. MFG‐E8 can be secreted bound to the surface of EVs [104] and in this format may be ready for interaction with α_v_β_3_ and α_v_β_5_ integrins of target cells, as described for corpse clearance [105]. ApoEVs may act as ligand foci for target cell receptors, a case in point being the PtdSer‐binding bridging molecules GAS6 and Protein S required for TYRO‐AXL‐MER family receptor tyrosine kinase signalling, not least MER [82, 92], which, as stated earlier, is pivotal in anti‐inflammatory signalling during corpse clearance, like MFG‐E8 [106]. A further example is the phospholipid‐binding protein Annexin A1, which undergoes caspase‐dependent translocation from the cytosol to the external leaflet of the plasma membrane, where it associates with PtdSer and acts as a ligand for efferocytosis [107]. Annexin A1 has been reported as a marker of MVs [55] and as a mediator of anti‐inflammatory signalling [108]. Detailed cell targeting mechanisms await elucidation, but we anticipate multiple mechanisms will be shown to be dependent on diverse factors, from the membrane topology of individual ApoEVs to the lineage and pathophysiological status of their recipient cells.

## Functional attributes of ApoEVs

Although the relative importance of any of the candidate ApoEV receptors and pathways underlying responses of phagocytes or indeed any target cell type remains unknown, burgeoning evidence indicates that ApoEVs trigger diverse responses in recipient cells. In line with the known properties of apoptosis, it seems likely that ApoEVs, especially ApoBDs, participate in the recycling and repurposing of the components of phagocytosed apoptotic cells as occurs, for example, in the fuelling of anti‐inflammatory and pro‐resolution reprogramming of macrophages [109, 110], of continued efferocytosis [111], and of proliferative capabilities of the phagocyte population [112]. The pro‐resolution properties of efferocytosis are among the most renowned features of apoptosis, as illustrated by the production of TGF‐β1, PGE_2_, and IL‐10. The latter is stimulated through the recycling of fatty acids [109], while TGF‐β1 is driven by PGE_2_ following CD36 signalling and via DNA methylation activated by the recycling of apoptotic cell‐derived methionine [110]. Cholesterol is another recycled component of apoptotic cells that stimulates phagocytes, in a cellular homeostatic response involving the PtdSer receptor BAI1, to increase cholesterol efflux via ABCA1 upregulation [113]. This pathway acts independently of a further pro‐resolution programme whereby cholesterol regulates ABCA1 and cholesterol efflux downstream of LXR (the liver X receptor) [114].

### Immunomodulatory roles

Again, in accordance with the accepted properties of apoptotic cells, ApoEVs have been shown to modulate innate immune cells, through carriage of CX_3_CL1 (fractalkine) and ICAM‐3, which act as chemoattractants for mononuclear phagocytes [115, 116]. Intriguingly, proof‐of‐principle studies indicate that a wide spectrum of functional chemokines may be carried by ApoEVs by way of their ability to bind to exposed PtdSer [80], raising the possibility that ApoEVs have broad functional potential in controlling chemokine signalling. ApoEVs carrying CX_3_CL1 can enhance efferocytosis via induction of MFG‐E8 [117] and may also suppress inflammatory responses in macrophages – as can MVs from healthy cells – through MER signalling [92, 118, 119]. However, in line with known immunogenic properties of apoptosis [120] in addition to its more classical anti‐inflammatory/tolerogenic features, the immunomodulatory activities of ApoEVs are complex and include immunostimulatory and pro‐inflammatory functions in certain contexts. As we have discussed, ApoEVs are rich in autoantigens, and it has long been known that ApoEV surfaces can be autoantibody‐reactive [61, 64], properties highly suggestive of their playing key roles in autoimmune disease pathogenesis, particularly under conditions of impaired efferocytosis. The preferential loading of at least some ApoEVs with autoantigens, notably DNA and other chromatin components, is presumed to contribute – since this is the case with apoptotic cells – to their efficient clearance by phagocytes, thereby helping to prevent autoimmune disorders. Conversely, autoantigen exposure, through immune complex formation with autoantibodies, also contributes to autoimmune disease aetiology [121]. There is no doubt that ApoEVs can indeed be efficiently engulfed by phagocytes [45], but the identity of the phagocyte is important in determining the outcome of the response: PtdSer may drive anti‐inflammatory responses in macrophages; by contrast, EV‐associated DNA may be highly pro‐inflammatory or immunogenic. For example, DNA carried by exosomes or exosome‐like EVs originating from tumour cells responding to chemotherapy (though not dying by effector caspase‐dependent apoptosis) is sensed by the cGAS‐STING (Stimulator of INterferon Genes) pathway in recipient dendritic cells that are consequently activated to promote anti‐tumour T cell‐mediated immunity [122]. ApoEVs from apoptotic lymphocytes have been shown to carry immunostimulatory DNA and can activate human plasmacytoid dendritic cells to produce interferon (IFN)‐α [123]. Additional mechanisms militate against the immunogenic nature of the chromatin content of ApoEVs, one being the activity of the secreted deoxyribonuclease, DNASE1L3, which digests chromatin at the surface of ApoEVs, rendering it immunologically silent [124]. ApoBDs from endothelial cells can generate sterile inflammation through carriage of IL‐1α [125]. Moreover, apoptotic endothelial cell‐derived exosome‐like small ApoEVs have been shown to stimulate autoantibody production, inflammation, and acceleration of aortic allograft rejection via activation of their 20S proteasome core cargo [126]. These EVs are also enriched in immunogenic RNAs, which have the potential to activate RIG‐I‐like receptors and toll‐like receptors [127]. The production of small, exosome‐like ApoEVs with pro‐inflammatory properties has also been reported for HeLa and other cancer cell lines [41]. Taken together, the available evidence indicates that, depending on context (including cargoes, originator cells, recipient cells, and processing pathways), ApoEVs have the capacity to activate diverse inflammatory, anti‐inflammatory, immunostimulatory, and tolerogenic responses. More work is required to dissect the underlying molecular cell biology.

### Effects on homeostatic programmes

In recent years it has become clear that, in addition to its roles in inflammation and immunity, apoptosis has wide‐ranging homeostatic effects in development, tissue turnover, remodelling, damage repair, and regeneration. Thus, apoptotic cells can engender proliferation (also known as compensatory proliferation, apoptosis‐induced proliferation), death (apoptosis‐induced apoptosis), and other programmes of activation and differentiation of diverse cell types in their locale, including stem cells [128, 129, 130, 131]. Accruing evidence indicates that ApoEVs contribute to these processes. An early *in vitro* investigation indicated that macrophages, but not fibroblasts, were induced to undergo apoptosis in response to ApoEV treatment [132]. Conversely, a recent *in vivo* study using time‐lapse optical imaging of developing zebrafish epithelia demonstrated that caspase 3‐induced ApoBDs carrying Wnt8a from dying epithelial stem cells can activate proliferation of their neighbours [133]. In other studies, ApoEV preparations from cultured mature endothelial cells (which comprised a mixture of ApoBDs and mainly smaller ApoEVs) were shown to be capable of promoting proliferation and differentiation of endothelial progenitor cells and have potential, therefore, to be active in vascular repair [134]. Moreover, in atherosclerosis, ApoEVs prepared in the same way from endothelial cells elicited the production of vascular‐protective CXCL12 in recipient endothelial cells via transfer of miR‐126 [135]. Additional work has indicated that ApoEVs produced by neointimal smooth muscle cells not only are, by contrast, procoagulant and thrombogenic but also inhibit blood flow by directly inducing endothelial dysfunction (reduced vasodilation responses). Interestingly, this effect was abrogated by β_3_ integrin antagonists, suggesting that β_3_ integrins could be candidate receptors for ApoEVs on endothelial cells. In these studies, however, β_3_ integrin expression was also found on the ApoEVs [136]. Monocyte‐derived ApoEVs are also capable of inducing pro‐thrombotic changes in endothelial cells [137].

Recent work has implicated ApoBDs in the osteoclast–osteoblast communication that is required for bone remodelling. Unexpectedly, the active mechanism was found to require ‘RANKL (receptor activator of NF𝜅B ligand) reverse signalling’ in osteoblasts stimulated through interaction with RANK on osteoclast‐derived ApoBDs [138]. In addition, ApoEVs from MSCs (albeit exogenously derived) have been reported to promote the healing of skin wounds and hair growth through Wnt/β‐catenin pathway activation [139]. The same group found that ApoEVs isolated from a mouse macrophage tumour cell line (RAW 264.7) using a similar protocol were taken up by MSCs and promoted adipogenesis but inhibited osteogenesis through transfer of miR155 [140]. ApoEVs prepared from cultured dental pulp stem cells in a similar way were found to exhibit pro‐angiogenic properties [141]. Further effects of ApoEVs in various pathologies are summarised in Table 1.

**Table 1:** Clinical applications of ApoEVs.

Disease	EV size and characterisation	Clinical application	Mechanism of action	Reference
Colon cancer and cancer stem cell renewal	Size range: 50–350 nm, characterised using NTA, TEM, and PET/CT imaging of *in vivo* EV uptake and biodistribution	EV‐based drug delivery of napabucasin to inhibit cancer stem cell activity.	Inhibition of cancer stem cell self‐renewal through reduced expression of STAT1 and CD44 in tumour tissues incubated with EV‐encapsulated napabucasin.	[163]
Heart failure	Size range: not assessed, characterised using FACS.	Differentiation between reduced ejection fraction and preserved ejection fraction heart failure	N/A	[164]
Heart failure	Size range: not assessed, characterised using FACS.	EV to mononuclear progenitor cell ratio used as a prognostic marker of all‐cause mortality, heart failure‐related death, or hospitalisation.	N/A	[165]
Cardiovascular disease and inflammation	Size range: not assessed, characterised using FACS.	Correlation between circulating EVs and IL‐6.	N/A	[166]
Myocardial infarction	Size 100–800 nm, characterised using WB, TEM, and DLS.	Myocardial repair following myocardial infarction.	Macrophage‐specific targeting of engineered neutrophil ApoEVs containing hexyl 5‐aminolevulinate hydrochloride enhanced efferocytosis and reprogramming to promote resolution of myocardial inflammation.	[167]
Cardiovascular health	Size not reported, characterised using FACS.	Acute exercise led to a reduction in ApoEVs and other EVs; greater in overweight inactive individuals.	N/A	[168]
Cardiac allograft vasculopathy	Size not reported, characterised using FACS.	Diagnostic biomarker for patients with allograft vasculopathy.	N/A	[169]
Coronary artery disease	Size not reported, characterised using FACS.	Diagnostic biomarker for impaired coronary vessel endothelial function.	N/A	[170]
Diabetes and insulin resistance	Size range: 65.5–584 nm, characterised using TEM, NTA, WB, and FACS of specific cargoes.	Reduced insulin resistance, improved glucose tolerance and lower levels of hepatic steatosis.	Macrophage reprogramming following enhanced efferocytosis of ApoEVs leading to an anti‐inflammatory macrophage phenotype.	[140]
Insulin resistance and chronic heart failure	Size range: not assessed, characterised using FACS.	Diagnostic and prognostic markers for insulin resistance in chronic heart failure and insulin resistance.	N/A	[171]
Hepatitis and diabetes	Size not reported, characterised using FACS.	Diagnostic biomarker in patients with chronic hepatitis C and type 2 diabetes using endothelial and platelet‐derived EVs, which correlate with oxidative stress and predispose to increased cardiovascular risk.	Increased oxidative stress in patients with chronic hepatitis C and type 2 diabetes, higher levels of endothelial and platelet‐derived EVs. EV number positively correlated with oxidative stress markers.	[172]
Glaucoma	Size range: defined as <1 μm, characterised using FACS.	Diagnostic biomarker for pseudoexfoliative glaucoma.	N/A	[173]
Haematology and coagulation	Size range 50–600 nm, characterised using NTA, TEM, mass spectrometry, FACS, protein quantification.	Fibrin generation.	ApoEVs and MVs more pro‐coagulant than endosome‐derived EVs.	[174]
HIV	Size range: not assessed, characterised using FACS.	Surrogate marker for T‐cell activation in HIV and levels of apoptosis in lentiviral infections.	N/A	[175]
Bone repair	Size 72–90 nm, characterised using TEM, WB, and NanoFCM.	N/A	Functionalised MSC EVs incorporated fibrin‐specific antibody within the EV membrane promoting fibrin retention in a rat model with a surgically acquired bone defect.	[176]
Osteoporosis	Size not reported, characterised using FACS.	Potential therapy to promote bone repair in osteopenia.	Loss of ApoEVs impaired MSC self‐renewal, which was reversed by exogenous addition of ApoEVs. MSC engulfment of ApoEVs via integrin α_v_β_3_. Reuse of ApoBD‐derived ubiquitin ligase RNF146 and miR‐328‐3p inhibited Axin1 and activated Wnt/β‐catenin pathway.	[97]
Wound healing	Size 104–194 nm, characterised using NTA, TEM, proteomics, WB and FACS.	N/A	Enhanced wound healing through transfer of SOX‐2 to MSCs, activating the Hippo signalling pathway.	[177]
Inflammation	Size 100–800 nm, characterised using SEM, TEM, WB, Zeta potential, DLS.	Potential therapeutic use of EVs to mediate systemic inflammation.	Chimeric ApoEVs constructed through conjugation of silica nanoparticles and ApoEVs loaded with miRNA‐21 or curcumin to target macrophages and modulate inflammation.	[178]
Renal disease, hypertension, and microalbuminuria	Size range: not assessed, characterised using FACS.	Diagnostic biomarker for elevated urinary albumin excretion.	N/A	[179]
Renal disease, hypertension, and microalbuminuria	Size range: not assessed, characterised using FACS and microparticle concentration/μl.	Prognostic decline in glomerular filtration rate assessing endothelial apoptotic microparticles and endothelial progenitor cell ratio.	N/A	[180]
Female factor infertility and intrauterine adhesions	Size range 200–3,600 nm, characterised using NTA, TEM, protein quantification of EV.	Treatment of intrauterine adhesions and infertility secondary to this.	EV incorporation into hyaluronic acid promotes endometrial repair and reduced intrauterine adhesions leading to higher pregnancy rates.	[181]
Pre‐eclampsia	Size not reported, characterised using FACS.	Antenatal diagnostic testing for pre‐eclampsia through identification of DNA‐containing EVs; potential disease monitoring.	N/A	[182]
Systemic lupus erythematosus	Size range: 300–1,500 nm, characterised using FACS, electron microscopy.	SLE monitoring.	EVs induced pro‐inflammatory changes in circulating dendritic cells; enhanced neutrophil extracellular traps which drive anti‐chromatin autoimmune response in SLE.	[183]
Systemic lupus erythematosus	Size not reported, characterised using FACS.	Diagnostic biomarker for SLE.	EVs from patients with active SLE contain higher levels of acetylated chromatin compared to those in remission; they induce neutrophil extracellular traps and drive formation of reactive oxygen species.	[184]
Rheumatoid arthritis	Size 100 nm, characterised using TEM, WB, and proteomics.	N/A	M2 macrophage‐derived EVs reprogrammed M1 macrophages within joints to quiescent, anti‐inflammatory M2 state leading to reduced inflammation.	[185]

DLS, dynamic light scattering; FACS, fluorescence activated cell sorting (flow cytometry); nanoFCM, nano‐flow cytometry; NTA, nanoparticle tracking analysis; PET/CT, positron emission tomography/computed tomography; SEM, scanning electron microscopy; SLE, systemic lupus erythematosus; TEM, transmission electron microscopy; WB, western blotting.

Ample evidence now indicates that many cancers may evolve through ‘hijacking’ of homeostatic, apoptosis‐driven proliferative, repair, and regenerative tissue conditioning responses. It is noteworthy that aggressive cancers of diverse lineages tend to contain relatively high constitutive levels of tumour cell apoptosis [142, 143]. For example the rate of cell loss is ~70% of that of cell gain in Burkitt's lymphoma [144], and in glioblastoma up to 70% of the overall tumour cell population is apoptotic [66]. In lymphoma, constitutive apoptosis was found to drive tumour growth, angiogenesis, and a reparatory macrophage activation state in mouse models [145]. Anti‐cancer therapy‐induced apoptosis appears able to promote tumour cell repopulation and growth via a PGE_2_‐dependent pathway activated by caspase 3 [146, 147, 148]. That ApoEVs contribute to oncogenic effector mechanisms has been demonstrated in glioblastoma. Thus, ApoEVs participate in an intercellular signalling pathway that fosters the acquisition of an aggressive phenotype in glioblastoma clones. Key ApoEV cargoes were found to be components of spliceosomes, notably RBM11, which endowed recipient cells with greater proliferative, migratory, and anti‐cancer drug resistance capacities by favouring expression of more oncogenic isoforms of MDM4 and Cyclin D1 [66]. The migratory capacity of triple‐negative breast cancer and non‐small cell lung cancer cell lines has also been shown to be enhanced by ApoBDs operating via PtdSer‐GAS6‐AXL signalling [149].

These observations are in line with the behaviour of EVs from healthy cells, which have functions in promoting oncogenic evolution through horizontal RNA and protein transfer [150, 151]. Horizontal transfer of gDNA by healthy cell‐derived EVs has also been demonstrated and has implications in cancer biology and normal physiology through its ability to affect development and variety in cell populations [67, 70, 152]. The capacity of ApoEVs to transfer gDNA in this way remains unresolved, but it has long been known that apoptotic cell corpses (probably in mixtures with relatively large ApoBDs) are capable of horizontally transferring oncogene‐carrying gDNA (and also viral DNA) to efferocytes such as macrophages, dendritic cells, fibroblasts, endothelial cells, and smooth muscle cells [153, 154]. Although transferred DNA may be degraded in normal cells, a potential pathway being via Chk2‐, p53‐, and p21‐dependent sensing of DNase II‐fragmented DNA [155], mechanisms for the persistence of functional apoptotic cell‐derived gDNA have been demonstrated, notably through suppression of p53 and Rb [154]. Therefore, in cancer pathogenesis, pro‐oncogenic contributions from apoptosis may extend to DNA transfer, and the propensity for ApoEVs to carry gDNA cargo suggests that ApoEV‐mediated horizontal transfer of gDNA carrying oncogenic mutations has the potential to manipulate the oncogenic potency both of transformed cancer cell populations and of their supporting cells in the tumour microenvironment.

## Clinical applications of ApoEVs

Growing understanding of the increased release of EVs in various disease states and differential loading of cargoes has led to a revolution in their use as novel diagnostic and prognostic markers [156, 157]. The innate ability of EVs to deliver these differentially expressed cargoes to recipient cells, manipulate the microenvironment, and alter tissue function has led to EVs themselves becoming therapeutic targets and novel vehicles for drug delivery [158]. Unmodified EVs are now under investigation for their potential therapeutic efficacy in several clinical trials including drug‐resistant infection (https://clinicaltrials.gov/ct2/show/NCT04544215), wound healing (https://clinicaltrials.gov/ct2/show/NCT05475418?term=exosomes%2C+extracellular+vesicles&draw=2&rank=3), and neuroprotection in pre‐term infants (https://clinicaltrials.gov/ct2/show/NCT05490173?term=exosomes%2C+extracellular+vesicles&draw=2&rank=25), to name but a few [159]. Furthermore, EVs hold diagnostic promise; of particular note is the groundbreaking discovery of the potential for EV surface markers like Glypican‐1 to be used in early cancer detection [160]. Most EVs under investigation for therapeutic efficacy are those produced by healthy cells, in some cases loaded with pharmacological agents; few have focused on the utility of ApoEVs. This may be due in part to the outdated view that ApoEVs are a passive byproduct of cell death rather than a physiologically relevant feature of the highly regulated and coordinated apoptosis programme [161]. ApoEVs also have promising clinical applications. They are produced over a broad size spectrum, and the larger subtypes of ApoBDs possess great cargo‐carrying capacity. While the identification of specific markers of ApoEVs remains a challenge, the criteria used to distinguish ApoEVs from EVs produced by healthy cells varies substantially in the literature. Thus, EV heterogeneity and contamination of ApoEV subtypes remain a problem (Figure 1). Despite these issues, there is a rapidly growing interest in the functional contributions of ApoEVs to a range of pathologies and in their utility as either diagnostic or prognostic biomarkers (Table 1).

Manipulation of cargo loading, exogenous incorporation of molecules within EVs, or the creation of synthetic EVs to harness their wide distribution and low immunogenicity have long been of interest to numerous researchers in the EV field. While reliable EV‐based therapeutics are still in their infancy, a growing number of wide‐ranging applications have been developed, including EV‐based vaccines for infectious disease and cancer, immunosuppression and modulation in autoimmune encephalomyelitis, and delivery of siRNA to the central nervous system in patients with Alzheimer's disease [156]. As with all systemically delivered therapeutics, unwanted off‐target effects can limit the utility of the drug, and, given the wide biodistribution of EVs, this has the potential to limit their clinical utility in the future. However, given the rise of precision medicine and a move from generic treatments to patient‐ and disease‐specific interventions, EVs are a novel and exciting area for future exploitation, and ApoEVs are no exception [162].

## Conclusions and future perspectives

Although ApoEVs remain the ‘third cousin’ in the EV family, behind exosomes and MVs of healthy cells, they are firmly established as important biological entities, released during the active programme of apoptosis. They are produced in a variety of functional ‘flavours’, which, depending on their apoptotic cell of origin and tissue context, can extend the legacy of that cell beyond its death, both in time and space. Significant advancements in our knowledge of ApoEVs have been made in recent years, and the stage is set for improving our understanding of all aspects of ApoEV biology, from their heterogeneity and the molecular mechanisms that underpin their production, cargo loading, and release to downstream mechanisms of cell targeting, recipient intracellular processing, signaling, and functional modulation. To dissect further the detailed molecular cell biology of ApoEVs, it will be important to standardise their classification by establishing that EVs under study are derived specifically as a consequence of the apoptosis programme and that certain minimal standards of definition of ApoEVs are met [23]. Significant advancements in ApoEV biology will undoubtedly be gained from comparative investigations that include the modulation of the apoptosis programme along with the application of novel technologies such as microfluidics [186] and optical imaging, both intracellularly [56] and in model organisms [187] producing ApoEVs. Single‐EV analytical approaches will be groundbreaking.

The large variety (and potential redundancy) of receptors involved in the interactions of apoptotic cells with their phagocytes, both professional and non‐professional, have long puzzled researchers in the field. Cell and tissue context‐specific roles for the multiplicity of soluble and phagocyte surface receptors are likely. Furthermore, individual receptors subserve distinct functions such as tethering of apoptotic cells (e.g. CD14 [188]) or delivering specific response signals from apoptotic‐cell interaction (e.g. MER, which can elicit differential signalling pathways, either for efferocytosis or, through a different cytoplasmic domain and depending on the co‐operation of TIM4, for proliferation [189]). We suspect that ApoEVs will prove to exert their biological effects, at least in part, through many of the ‘corpse clearance’ receptors and thereby help to rationalise the wide range of receptors involved in sensing apoptosis, both in phagocytes and non‐phagocytes, in order to generate downstream signals for divergent responses of recipient cells, from recycling to reprogramming (Figure 2). In this way, ApoEVs are likely participants in the integration of signals that determine the varied responses to apoptosis, complementing the apoptotic cells themselves. It is tempting to speculate that ApoEVs can impart fundamental effects of apoptosis in addition to those we have discussed, akin to the imprinting of innate immune memory that accompanies efferocytosis [190].

In the widening arena of EV research, many important questions remain, and there is much scope for advancing our understanding of ApoEV biology and its applications. For example: What are the mechanisms of selective cargo loading into ApoEVs? How do small, exosome‐like ApoEVs arise? How are ApoEV cargo components protected from degradation in recipient cells? What are the programmes of responses of recipient cells of various lineages in health and disease? Much is likely to be gained through resolving the heterogeneity problems and from an understanding of the multiple controls on cells and tissues that impinge on EV production and release, including additional regulated cell death modalities, for example necroptosis, in which EVs are also produced [191]. In these contexts, it is remarkable that endothelial cells with active autophagic and apoptotic programmes appear to release ApoEVs with unique cargoes, notably ATP, mitochondria, and endoplasmic reticulum, but lacking nuclear material. Although release of these ApoEVs was found to be caspase‐dependent, it was independent of ROCK1 [192]. Production of immunogenic exosome‐like ApoEVs may be critically dependent upon autophagic activity [193], and autophagic components are important for selective EV loading of RNA‐binding proteins [194], which are common cargoes of ApoEVs. Finally, as well as intercellular communication, the tissue activities of ApoEVs may extend to regulating the extracellular matrix through degradation and remodelling by key cargoes such as MMPs (just as EVs from healthy cells [195]), especially since apoptotic cells can produce and process MMPs [145]. There seems little doubt that the future holds much promise for ApoEVs, not only from the perspective of their biology in normal homeostasis and in disease pathogenesis but also in relation to their potential application in diagnostics and therapeutics.

## Author contributions

CDG and MPR conceived, planned, wrote and edited this review.
